# Emerging Rabbit Hemorrhagic Disease Virus 2 (RHDVb), Australia

**DOI:** 10.3201/eid2112.151210

**Published:** 2015-12

**Authors:** Robyn N. Hall, Jackie E. Mahar, Stephanie Haboury, Vicky Stevens, Edward C. Holmes, Tanja Strive

**Affiliations:** CSIRO Health and Biosecurity, Canberra, Australian Capital Territory, Australia (R.N. Hall, J.E. Mahar, S. Haboury, T. Strive);; Invasive Animals CRC, Bruce, Australian Capital Territory, Australia (R.N. Hall, S. Haboury, T. Strive);; The University of Sydney, School of Biological Sciences, Sydney, New South Wales, Australia (J.E. Mahar, E.C. Holmes);; CSIRO Australian Animal Health Laboratories, Geelong, Victoria, Australia (V. Stevens)

**Keywords:** rabbit hemorrhagic disease virus, RHDV2, RHDVb, biocontrol, viruses, Australia, zoonoses

**To the Editor:** In May 2015 an isolate of the recently emerged variant of rabbit hemorrhagic disease virus (RHDV), RHDV2, was identified in an Australian wild rabbit (*Oryctolagus cuniculus*). RHDV2 (also called RHDVb) was first described in outbreaks in France in 2010 (*1*), then Italy and Spain in 2011 (*2*,*3*) and in Portugal from 2012 onwards (*4*). The virus is a genetically and antigenically distinct variant of RHDV that is able to partially overcome immunity to classical strains of RHDV (*1*,*2*). In contrast to case-fatality rates for other strains of RHDV, those for RHDV2 infection have been reported to be lower in mature rabbits (0%–75% in 1 study, compared with >90% for classic RHDV infection) (*3*) but higher (50% in 1 study) in rabbit kittens as young as 30 days of age, which are normally highly resistant to lethal RHDV infection (*2*). RHDV2 has been reported to spread effectively in domestic rabbits in Europe (*3*); it may be replacing existing strains of RHDV that infect wild rabbits on the Iberian Peninsula (*5*), possibly because of its ability to partially overcome immunity to these strains.

As part of ongoing opportunistic surveillance of RHDV field outbreaks, we analyzed 3 isolates from dead adult wild rabbits found in the wider Canberra region of Australia. The first virus isolate (BlMt-1) came from a rabbit found in Australian Capital Territory on May 13, 2015. The second isolate (BlueGums-2) was taken 3 days later from a rabbit in New South Wales, 50 km north of Canberra. On June 9, another dead rabbit, from which the third isolate (BlMt-2) was obtained, was found in the same location as the first. The isolates were initially typed by amplifying and sequencing the capsid gene (*6*), and the results were confirmed independently in 2 laboratories. Subsequently, full-length genome sequencing of the 3 virus isolates was performed by amplifying the viral genomes in overlapping fragments (*6*); the fragments were then sequenced by using Illumina MiSeq technology (*7*).

Phylogenetic analysis revealed that 2 isolates, BlMt-2 and BlueGums-2, were closely related to field strains currently circulating in Australia (*7*) (Figure). Strikingly, the third isolate (BlMt-1) was most closely related to an RHDV2 variant generated by recombination of the RHDV2 capsid gene (Figure, panel B) and the RHDV genogroup 1 nonstructural genes (Figure, panel A), which has recently been reported to be circulating in Portugal and the Azores (*8*,*9*). How the virus variant arrived in Australia is unclear, although our analysis indicates that it likely originated in southern Europe. 

**Figure:**
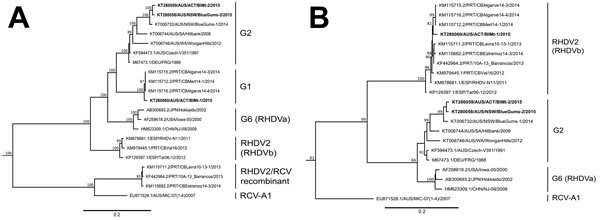
Maximum-likelihood phylogenetic analysis of the nonstructural protein genes (A) and the capsid gene (B) of rabbit hemorrhagic disease virus (RHDV) sequences. The 3 recent Australian field isolates sequenced for this study (indicated in bold) were aligned with representative RHDV and Australian rabbit calicivirus (RCV-A1) sequences from GenBank (accession numbers indicated in taxa names). Phylogenetic analysis was conducted separately for both the nonstructural genes (panel A) and the capsid gene (panel B). Phylogenies were rooted by using an early European brown hare syndrome virus strain (not shown). Statistical support for individual nodes was estimated from 1,000 bootstrap replicates with values shown for only those nodes where the bootstrap support was ≥70% (and all major nodes). Phylogenies were constructed by using the general time reversible plus gamma model of nucleotide substitution, as determined in jModelTest, by using PhyML(as available in Geneious version 8.1.5; Biomatters Limited, Auckland, New Zealand). Scale bars are proportional to the number of nucleotide substitutions per site.

In 1991, CSIRO imported the Czech351 strain of RHDV to assess its potential as a biocontrol tool for controlling the European rabbit, which causes massive economic and ecologic damage and is a declared a pest species in Australia. In 1995, after initial testing in quarantine, the virus escaped during field trials being conducted on a coastal island through passive fly transmission and subsequently spread across the continent. The RHDV2 variant reported here has not previously been investigated by CSIRO, and the organization did not possess it.

Rabbits are found in ≈70% of the 6.7 million km^2 ^Australian continent and Tasmania. However, natural outbreaks of RHDV infection are monitored in comparatively few locations, and their detection largely relies on opportunistic sampling. To follow the spread of this new variant and determine its current range, increased surveillance of outbreaks of RHDV infection in both wild and domestic rabbits in Australia is urgently required. The unique traits of strain RHDV2, particularly its ability to overcome immunity to classical RHDV strains (including vaccine strains) (*3*) and to infect rabbits at a younger age (*2*), may have wide-ranging implications for rabbit biocontrol in Australia. In parallel with similar efforts in Europe, strategies need to be developed to protect commercial and pet rabbits.

Tracking the spread of RHDV2 in Australia, in competition with existing field strains, highlights the value of Australia’s rabbits and their diseases as a model system for emerging infectious diseases. The point releases of both myxoma virus and RHDV into large naive host populations represent a grand experiment in disease emergence and evolution (*10*), which provides a unique opportunity to study the virulence evolution of emerging pathogens as well as their complex interactions with each other. It is notable that since the release of RHDV in Australia in 1995, strains of 1 viral lineage dominate the viral population nationwide despite hundreds of deliberate re-releases of the original virus strain for local rabbit control, which strongly suggests it has a major selective advantage (*7*). That RHDV2 appeared in a wild rabbit is therefore remarkable, particularly because Australian field strains were spreading simultaneously in the same area. Comparing the epidemiology of this strain in Australia to the epidemiology of its well-documented spread in Europe will provide valuable insights into RHDV epidemiology relevant to both continents.
